# Phytochemicals: potential alternative strategy to fight *Salmonella enterica* serovar Typhimurium

**DOI:** 10.3389/fvets.2023.1188752

**Published:** 2023-05-16

**Authors:** Abdulaziz M. Almuzaini

**Affiliations:** Department of Veterinary Medicine, College of Agriculture and Veterinary Medicine, Qassim University, Buraydah, Saudi Arabia

**Keywords:** *Salmonella*, antimicrobial resistance, plant extract, immune response, livestock

## Abstract

The rise of multidrug resistant (MDR) microorganisms is a great hazard worldwide and has made it difficult to treat many infectious diseases adequately. One of the most prevalent causes of outbreaks of foodborne illness worldwide is *Salmonella*. The ability of this and other harmful bacteria to withstand antibiotics has recently proven crucial to their effective control. Since the beginning of time, herbal medicines and phytochemicals have been employed for their potent antibacterial action and there is a growing trend toward the production of plant based natural products for the prevention and treatment of pathogenic infections. Numerous phytochemicals have been proven effective against the molecular determinants responsible for attaining drug resistance in pathogens like efflux pumps, membrane proteins, bacterial cell communications and biofilms. The medicinal plants having antibacterial activity and antibiotics combination with phytochemicals have shown synergetic activity against *Salmonella enterica* serovar Typhimurium. The inhibitory effects of tannins on rumen proteolytic bacteria can be exploited in ruminant nutrition. Improved control of the rumen ecology and practical use of this feed additive technology in livestock production will be made possible by a better knowledge of the modulatory effects of phytochemicals on the rumen microbial populations in combination with fermentation. This review focuses on the development of antibacterial resistance in *Salmonella*, the mechanism of action of phytochemicals and the use of phytochemicals against *S. enterica* serovar Typhimurium. The advances and potential future applications of phytochemicals in the fight against resistant are also discussed.

## Introduction

Foodborne diseases are brought on by consuming food, herbs, and beverages that have been contaminated by microorganisms as well as hazardous compounds including heavy metals (1), mycotoxins and bacterial toxins, as well as fermentation byproducts including biogenic amines and ethyl carbamate (2). Most of these foodborne illnesses are a problem for worldwide public health because they are brought on by pathogenic bacteria, viruses and parasites (3, 4). One of the main causes of foodborne diseases is Salmonellosis infection, which is brought on by a species of *Salmonella* (5, 6). *Salmonella* has long been recognized as an important zoonotic pathogen of economic importance in animals and humans. *Salmonella enterica* serovar Typhimurium can infect a wide range of animal species, e.g., cattle, sheep, goats, pigs, horses and poultry (7). There are pathovariants within serovar Typhimurium that are host-adapted, including sequence type (ST) 313 (8, 9) linked to invasive NTS (iNTS) in humans in sub-Saharan Africa and definitive phage types (DT) 2 and DT99 in pigeons (10), DT40 and DT56 in passerine birds (11) and U288 in pigs (12).

*Salmonella* Typhimurium belongs to the *Enterobacteriaceae* family. These Gram negative, flagellated rods are facultative anaerobic and do not produce spores (13). *S*. Typhi, which causes typhoid fever and gastrointertitis, a multi systemic disease, is a public health concern in developing countries. *Salmonella* species can be found throughout nature, although their primary sources include the GIT of mammals, reptiles, birds, and insects, as well as the environment that has been contaminated by human or animal waste (14). The most common clinical manifestation of salmonellosis in animals is an enteric disease, but numerous other conditions may be observed including acute septicemia, abortion, arthritis and respiratory diseases (15, 16).

Antibiotics are used in food animal production to promote growth and to prevent, treat and control infectious diseases. The antibiotics chloramphenicol, trimethoprim-sulfamethoxazole, ampicillin, fluoroquinolones and cephalosporin are the treatment options for *S*. Typhimurium (17). In emerging and particularly underdeveloped nations, the rise in antibiotic resistance in this disease has been a major concern. Resistance to antimicrobial agents may be defined as is the inability of bacteria to respond to medications that were once thought to be useful in treating infections brought on by that particular pathogen (18). By absorbing foreign DNA or by mutating its own DNA, *S*. Typhimurium can develop antibiotic resistance (19). Resistance to these antibiotics in *S*. Typhimurium strains is known as multi drug resistance (MDR) (20, 21). The rapid emergence of MDR among bacteria is caused by ongoing selective pressure and the evolution of new bacterial survival mechanisms in response to commonly used or recently produced antibiotics (22). Like all bacteria and depending on the strain and external factors, *Salmonella* attach to a variety of biotic and abiotic surfaces and form biofilms, posing a concern in food sectors and healthcare settings (23, 24). Biofilms are linked to about 80% of all bacterial illnesses in humans (25). Thus, *Salmonella* species discovered in their planktonic phase are typically susceptible to being eliminated by disinfectants or antibiotics, are significantly more resistant to these actions in biofilms (26). However, it costs a lot of money and time to find new antibiotics, and it takes around 10 years to get a new antibiotic on the market (27).

Therefore, there is a great effort to tackle antibacterial resistance and create effective, ecofriendly, and safe anti-biofilm techniques as well as therapeutic methods (28). Natural compounds, especially those derived from plants, have been an essential source of therapeutic medications over the past years with distinct features that make them suitable for use as alternative treatments for MDR infections that pose medical challenges (29). In order to protect themselves from microbial, herbivore, and insect predators, plants have an almost infinite capacity to mix aromatic molecules, primarily phenolic compounds, polyphenols, alkaloids, flavonoid, terpenoids, ketones, and essential oils (30). Many bioactive substance derived from substances, known as phytochemicals have been studied and found to be relatively safer than synthetic counterparts (31). These compounds also exert various therapeutic effects due to their high potency (32). Phytochemicals also known as phytobiotics or phytogenics that are added to animal feed to increase production. Phytochemicals are also proposed for use as antioxidants in animal feed, which will protect animals from oxidative damage caused by free radicals (33, 34). These phytochemicals have a variety of mechanisms of action, including the inhibition of efflux pumps and target altering and drug degrading enzymes (35). When used alone or in combination with other antibiotic compounds, phytochemicals have been found to have antimicrobial activities against clinically significant pathogens like *Salmonella* species, lowering the risk of developing a variety of diseases (36, 37). A successful method for modifying resistance is to use antimicrobial agents and phytochemicals in combination that will eliminate the resistance mechanism and still allow the medicine to be effective against resistant microorganisms (38). Plant extracts can be used to make natural additives with antibacterial properties that can be added to animal feed in an effort to reduce the use of antibiotics and switch to a more natural diet for animals (39). The main challenges that prevent plant based bioactive chemicals from being used commercially include a lack of raw materials, poor stability, high production costs, an unclear mode of action, and a lack of efficient regulatory systems (40). The aim of this review is to comprehensively present antibacterial resistance in *Salmonella*, the mechanism of action of phytochemicals and the use of plant-derived medicinal plants against *S*. Typhimurium.

## Antibacterial resistance in *Salmonella*

*Salmonella*, that is multi drug-resistant, has emerged as one of the major foodborne pathogens, threatening global public health safety (41). Antibiotics are used as feed supplements at sub therapeutic doses to the economic effectiveness of animal production, to enhance growth and feed conversion efficiency and to avoid diseases (42). However, using in feed antibiotics (IFAs) could result in the emergence of antimicrobial resistance as animal farming intensifies, posing a potential risk to human health (43).

*Salmonella* resistance has been reported to a wide variety of antibiotics including sulfamethoxazole, tetracycline, cefotaxime chloramphenicol, compound trimethoprim, ampicillin, cephalosporins and nalidixic acid (44, 45). It is well known that the development of biofilms results in a high level of resistance in the bacteria as well as the horizontal transmission of resistance between bacterial cells through transformation and conjugation (46, 47). The activity of efflux pumps, target adaptation, enzymes expressions and mutation are the antimicrobial resistance mechanisms that occur in planktonic cells (48).

### Mechanism of action of phytochemicals

Phytochemicals have possible biological effects, including antibacterial, antiviral, antioxidant, and anti-inflammatory, and used for animal nutrition and health improvement (43, 49, 50). Phytochemicals inhibit the growth of *S*. Typhimurium by several mechanisms (51). These might include preventing the bacterial attachment to host cells (52), reduction in the bacterial ability to produce proteins, cell wall, and nucleic acids (53), loss of the transmembrane electrochemical gradient and reduction of the osmoregulation of bacteria and increased nitric oxide (NO) synthesis, which has a deadly effect (54). Additionally, phytochemicals influence the immune system through immunomodulatory effects such as enhanced immune cell proliferation, modification of cytokines as well as higher antibody titers (55, 56).

### Inhibition of cell wall synthesis

**Figure 1:**
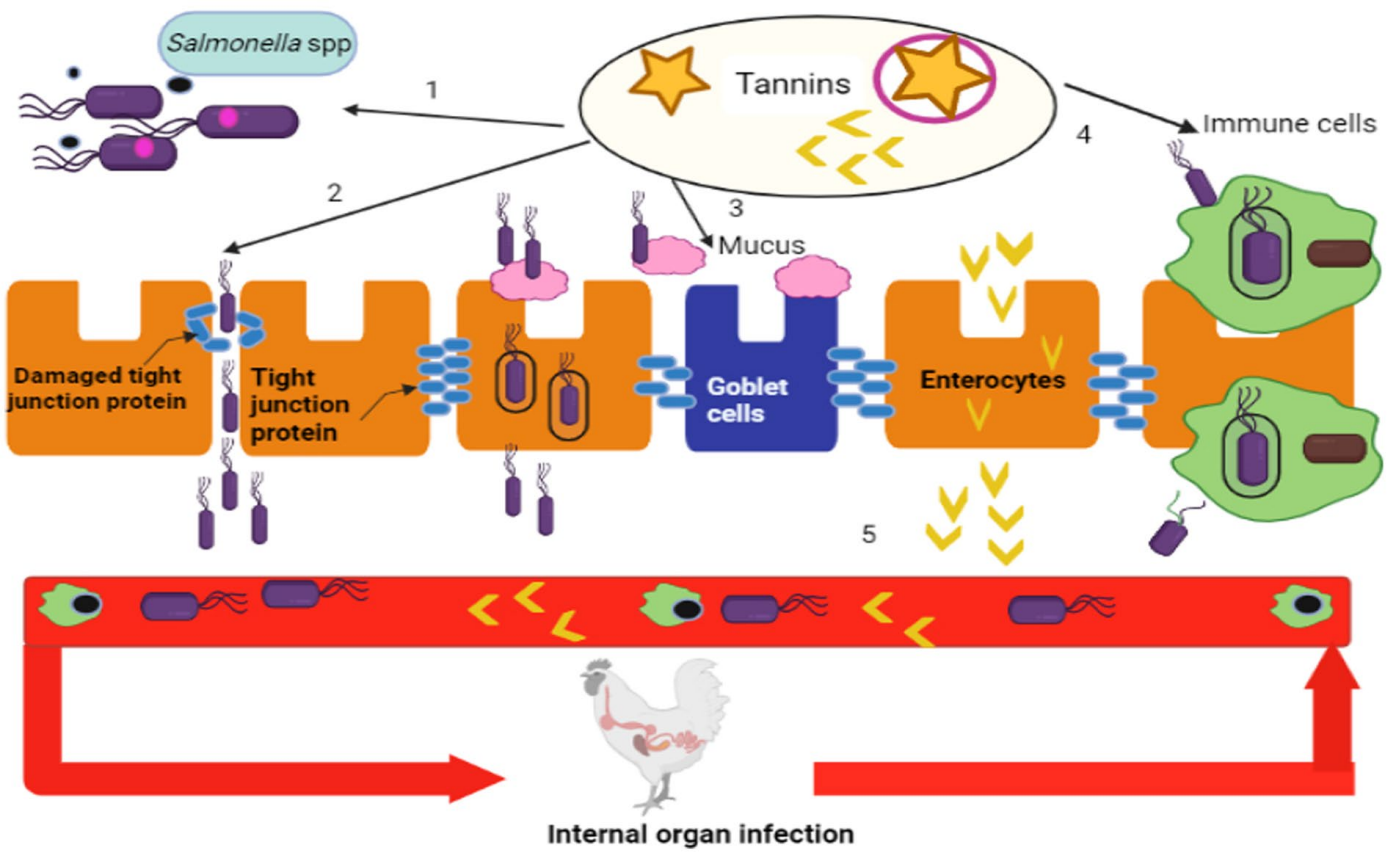
Systemic infection routes of *Salmonella* spp. and potential mechanisms of antibacterial actions of tannins (Retrieved from bio render).

N-acetylglucosamine (NAcGlc) and N-acetylmuramic acid (NAcMur) residues are repeated units that make up peptidoglycan and these repeating units are joined by short amino acid chains. The arrangement of amino acid residues is essential for giving bacteria strength and consequently protection (57). In order to better control the formation of the bacterial cell wall, phytochemicals have been found to be helpful in therapeutic approaches. Due to their impact on the bacterial cell wall, flavonoids have a marked antibacterial effect against a variety of bacterial and infectious diseases. The presence of more lipophilic flavonoids may also disrupt bacterial membranes (58). The lysis of cell walls has also been notice in bacteria exposed to phenolic mixtures. By targeting bacterial cell wall, tannins have qualities that inhibit the growth and protease activity of ruminal bacteria and if they are highly lipophilic, they also disrupt cell layers (59). The tannin of Sorghum has antibacterial activity against *S*. Typhi (60). Alkaloids often exert their antibacterial effects by intercalating themselves into the DNA and cell wall of bacteria (61). Through the upregulation of immunoglobulin A and mucin 2, tannins are helpful in maintaining chicken mucosal immune system components. Through paracellular and transcellular pathways, *Salmonella* spp. can enter the bloodstream and use immune cells to enter enterocytes, which are then dispersed throughout the muscles and organs of chickens. Tannins change the functions and expression of immune cells, mucus and tight junction proteins of chickens (62–64) as shown in Figure 1. Tannins inhibit the growth of *Salmonella* spp. in the intestine and decrease the quorum sensing of bacteria.

Tannins that are used against *S.* Typimurium is Condensed tannins from Quebaracho and *Calliandra calothyrsus*, Gallotannins from Tara and Sumach (Gall nuts), Flavanol gallates from Tea and *Acacia nilotica*, Tannic acid and Gallic acid. All of the tannins inhibited the growth of the *S.* Typimurium (65).

### Inhibition of bacterial physiology

When phytochemicals are added to the medium, the ensuing changes in membrane potential, inhibition of the function of membrane bound ATPase alter the physiological condition of the bacteria and metal ion chelation ultimately leading to bacterial death (66). The disruption of the membranes integrity by carvacrol, eugenol thymol and catechins has been observed to result in the release of cellular components and the ATP levels of cells (67). Additionally, terpinen-4-ol, 1,8-cineol, terpenes, alpha-terpineol and sesquiterpenes found in tea tree oil have the ability to alter membrane permeability, disrupt cell membranes, and inhibit cell development, leading to cell death in resistant organisms like *S. Typhimurium* (68).

### Inhibition of biofilms

**Figure 2:**
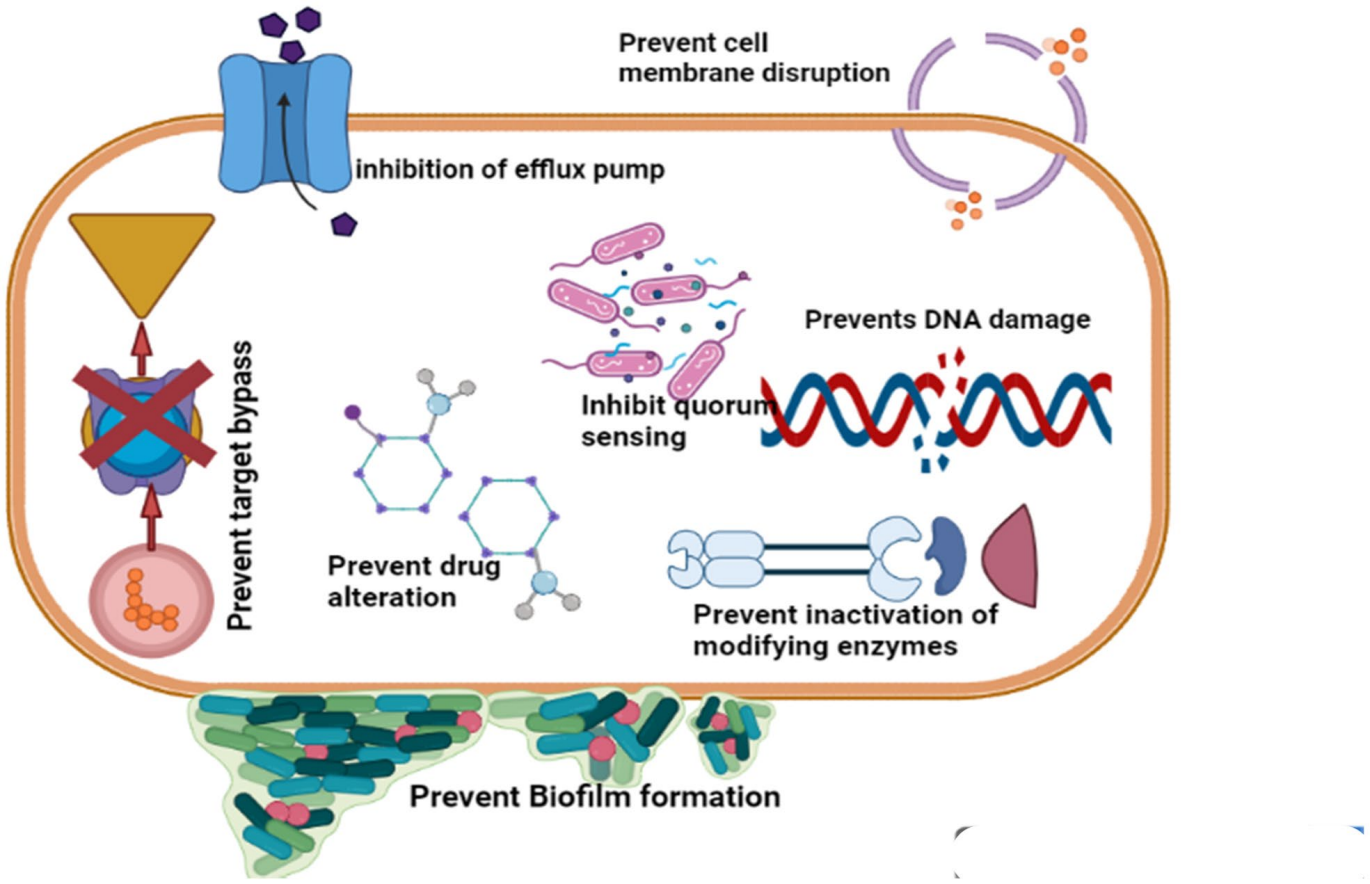
Phytochemicals are antibiotic alternatives and their mode of action (Retrieved from bio render).

Biofilm is a collection of microbial populations with surface integration that is enclosed in an exopolysaccharide matrix (69). Phytochemicals are employed to prevent and inhibit biofilm growth as well as to combat the development of antibacterial resistance, by taking advantage of their disruption of some of the key elements involved in the formation of biofilms, such as motility, attachment, intercellular accumulation and interaction (70, 71) shown in Figure 2.

Essential oils (EOs) components, lectins, alkaloids, polyacetylenes and polypeptides and terpenoids, phenolics, inhibit *Salmonella* growth and biofilm formation (72–74). The major ingredients in thyme oil and oregano, thymol and carvacrol, have antibiofilm properties against *S*. Enteritidis and *S*. Typhimurium on polypropylene (75). However, it has been demonstrated that *Salmonella* adapts to EOs and their constituents after being exposed to them at sub lethal concentrations by changing the expression of some important stress response genes. As a result, gains tolerance to both heterologous stressors and homologous (76). The anti-biofilm efficacy of two nutraceuticals of plant sources, *Andrographis paniculata* (Ap) and *Holarrhena antidysentrica* (Ha) are shown against *S*. Typhi biofilm development, whereby both exhibited and both showed antibiofilm and antimicrobial action by rupturing the membrane permeability of this pathogen (77).

### Synergistic phytochemicals as active site modification inhibitors

To mitigate the harmful effects of enteric infections, a number of phytochemicals are combined to create synergistic effects. Different resistance mechanisms such as increased activation of efflux pumps (EPs), expression of drug inactivating and target site modifying enzymes and modification of permeability barriers, can be neutralized by phytochemicals in combination with currently available antibiotics in a synergistic manner (78). Antibiotics and phytochemical substances have been given together to stop the emergence of resistance and it is effective tool for the management of MDR (79). For instance, ubiquitous phytochemicals from the barberry plant berberine and 5′-methoxyhydnocarpin exhibit synergism by accumulating inside bacteria and blocking the MDR pump (80). It has been discovered that streptomycin in combination with eugenol or cinnamaldehyde work synergistically to destroy the *S*. Typhimurium biofilm (81). Geraniol, bioactive compound that can be found in the essential oil of Helichrysum italicum, can restore the effectiveness of quinolones, chloramphenicol and beta lactam antibiotics against MDR bacteria (82). Studies on β-resorcylic acid, thymol, eugenol, carvacrol and trans-cinnamaldehyde revealed that they boosted *S*. Typhi DT104’s susceptibility to 5 antibiotics due to an inhibitory activity on EPs (83). The synergistic activity of phytochemicals with antibiotics shown in Table 1.

**Table 1:** Synergistic activity of phytochemicals with antibiotics and their minimum inhibitory concentration (MIC) or zone of inhibition (ZOI) values.

Plants	Antibiotic	Plant part	Extract	Biological activity	MIC/ZOI	Bacteria type	References
*A. sativum*	Ciprofloxacin	Bulbs	Methanolic	Inhibit efflux pump	27.5 ± 0.5 mm	*S*. Typhimurium NKS70	(84)
*S. aromaticum*	Ciprofloxacin	Flower buds	Ethyl acetate	Synergistic	23 ± 0.5 mm	*S*. Typhimurium NKS174	(84)
*R. cotinus*	Ciprofloxacin	Leaf	Methanolic	Synergistic	23.3 ± 0.5 mm	*S*. Typhimurium NKS773	(84)
*P. emblica*	Ciprofloxacin	Fruit	Ethyl acetate	Synergistic	27.5 ± 0.5 mm	*S*. Typhimurium NKS70	(84)
*B. aristata*	Tetracycline	Leaf	Methanolic	Synergistic	24.3 ± 0.8 mm	*S*. Typhimurium NKS70	(84)
*R. cotinus*	Tetracycline	Leaf	Ethyl acetate	Inhibit efflux pump	15 ± 0.1 mm	*S*. Typhimurium NKS773	(84)
*A. muricata*	Chloramphenicol	Leaves	Methanol	Anti-biofilm	12.5 μg/ml	*S*. Typhimurium ATCC 13311	(85)
Thymol	Amikacin	Fruit	Ethanol	Synergistic	0.5 μg/ml	*S*. Typhi ATCC 6539	(86)
Piperine	Kanamycin	Berry	Aqueous	Synergistic	2 μg/ml	*S*. Typhi ATCC 6539	(86)
Thymol	Streptomycin	Fruit	Ethanol	Synergistic	0.5 μg/ml	*S. enteritidis*	(86)
Thymol	Kanamycin	Fruit	Ethanol	Synergistic	0.5 μg/ml	*S*. Typhimurium	(86)
Thymol	Streptomycin	Fruit	Ethanol	Synergistic	8 μg/ml	*S*. Typhimurium	(86)
Thymol	Amikacin	Fruit	Ethanol	Synergistic	0.25 μg/ml	*S*. Typhimurium	(86)
Piperine	Kanamycin	Berry	Aqueous	Synergistic	1 μg/ml	*S*. Typhimurium	(86)
Piperine	Streptomycin	Berry	Aqueous	Synergistic	0.5 μg/ml	*S*. Typhimurium	(86)
Piperine	Amikacin	Berry	Ether	Bactericidal	1 μg/ml	*S*. Typhimurium	(86)
*W. somnifera*	Ciprofloxacin	Leaves	Methanol	Synergistic	27.5 ± 0.5 mm	*S*. Typhimurium NKS70	(87)
*Z. officinale*	Ciprofloxacin	Rhizome	Ethyl acetate	Synergistic	26 ± 0.7 mm	*S*. Typhimurium NKS70	(87)
*P. integerrima*	Ciprofloxacin	Leaves	Methanol	Synergistic	23.3 ± 0.5 mm	*S*. Typhimurium NKS773	(87)
*O. sanctum*	Tetracycline	Leaves	Methanol	Synergistic	32 ± 0.5 mm	*S*. Typhimurium NKS174	(87)
*M. charantia*	Tetracycline	Seeds	Ethyl acetate	Synergistic	18.5 ± 0.5 mm	*S*. Typhimurium NKS70	(87)
*C. asiatica*	Tetracycline	Whole plant	Methanol	Synergistic	28 ± 0.6 mm	*S*. Typhimurium NKS174	(87)
*P. latifolia*	Gentamicin	Leaves	Aqueous	Bactericidal	0.5 mg.ml	*S. enteritidis* ATCC 13076	(88)

### Plant- derived phytochemicals against *Salmonella*

Antibacterial resistance can be prevented, mitigated and reversed in a number of methods, whereby employing medicinal plant extracts with intrinsic antibacterial characteristics has been shown to be one of the most successful approaches (79, 89). When compared to synthetic chemicals, plant-derived antimicrobials have been found to be one of the most advantageous sources that are harmless due to their natural origin (90). For many years, bacterial infections have been treated by means of traditional healing systems using medicinal herbs (91). Around 80% of the developing nations uses traditional medicine made from phytochemicals as their primary health care modality (92, 93). Compared to their synthetic counterparts, medicinal plants are frequently less expensive, safer to use in terms of side effects and more accessible and also the probability for resistance development is most likely lessened due to the synergism of different bioactive compounds that can be present in plant-based formulation (any of them may belong to a different chemical group and be with a different mechanisms of action) (94). Gram-negative and Gram-positive bacteria are all affected by the bacteriostatic properties of resveratrol, which is a compound found in grapes and Itadori plants (95). Blackberry (*Rubus fruticosus*) and blueberry (*Vaccinium corymbosum*) pomace extracts were tested against *S*. Typhimurium at lethal and sub-lethal concentrations for their antibacterial, anti-motility, and antibiofilm activity. As growth promoters and to alter the gut microbiota, tannins and EOs are commercial food to a variety of domestic animal species (96). A commercial blend of phytonutrients that boosts innate immunity and lessens the harmful effects of enteric bacteria was approved in the Europe as the first botanical feed additive for enhancing the performance of broilers and livestock. This blend contains *capsicum oleoresin*, carvacrol and cinnamaldehyde (97). However, the best way to deal with antibacterial resistance is probably through a combinational strategy that allows for a synergistic interaction between plant extracts and conventional antibiotics. Streptomycin with either cinnamaldehyde or eugenol has been shown to work synergistically to destroy the *S*. Typhimurium biofilm (81). A detailed list of antibacterial activity of important medicinal plant extract and phytochemicals against *Salmonella* strains is provided in Tables 1, 2.

**Table 2:** Phytochemicals and their minimum inhibitory concentration (MIC) values against *Salmonella.*

Plants	Plant part	Extract	Biological activity	MIC	Bacteria type	References
*Cinnamomum verum*	Leaf	Aqueous	Antibacterial	0.1/0.013 v/v	*S*. Typhimurium	(98)
*Stereospermum kunthianum*	Leaf	Aqueous	Antibacterial	4.17 mg/ml	*Salmonella*	(99)
*Terminalia chebula*	Fruit	Aqueous	Inhibition of bacteria	15 mg/ml	*Salmonella*	(100)
*Rosa damascena*	Flower	Butanol	Antibacterial	62.5 μg/ml	*S*. Typhimurium	(100)
*Abutilon indicum*	Root	Chloroform	Bactericidal	0.6 mg/ml	*S*. Typhi	(101)
*Piper nigrum*	Seeds	Aqueous	Good inhibitory activity	>1,200 μg/ml	*S*. Typhimurium	(102)
*Aegle marmelos*	Leaf	Aqueous	Antibacterial	>6,000 μg/ml	*S*. Typhimurium	(103)
*Alstonia scholaris*	Leaf	Aqueous	Antibacterial	>5,000 μg/ml	*S*. Typhimurium	(103)
*Dalbergia latifolia*	Bark	Aqueous	Antibacterial	>5,000 μg/ml	*S*. Typhimurium	(103)
*Helicteres isora*	Root	Aqueous	Antibacterial	1,250 μg/ml	*S*. Typhimurium	(103)
*Oroxylum indicum*	Bark	Aqueous	Antibacterial	>5,000 μg/ml	*S*. Typhimurium	(103)
*Casuarina equisetifolia*	Root	Aqueous	Bactericidal	12–18 mm	*S*. Typhimurium	(104)
*Acacia mearnsii*	Bark	Acetone	Antibacterial	1.25 mg/ml	*S*. Typhimurium	(105)
*Aloe arborescens*	Leaves	Acetone	Antibacterial	2.5 mg/ml	*S*. Typhimurium	(105)
*Eucomis autumnalis*	Bulb	Acetone	Antibacterial	0.156 mg/ml	*S*. Typhimurium	(105)
*Hydnora africana*	Tuber	Acetone	Antibacterial	0.625 mg/ml	*S*. Typhimurium	(105)
*Pelargonium sidoides*	Root	Acetone	Antibacterial	0.312 mg/ml	*S*. Typhimurium	(105)
*Psidium guajava*	Leaves	Acetone	Antibacterial	1.25 mg/ml	*S*. Typhimurium	(105)
*Hypericum roeperianum*	Leaf	Acetone	Antibacterial	0.22 mg/ml	*S*. Typhimurium	(106)
*Bolusanthus speciosus*	Leaf	Acetone	Inhibitory activity	0.13 ± 0.04 mm	*S*. Typhimurium	(106)
*Elaeodendron croceum*	Leaf	Acetone	Inhibitory activity	0.26 ± 0.07 mm	*S*. Typhimurium	(106)
*Morus mesozygia*	Leaf	Acetone	Inhibitory activity	0.16 ± 0.11 mm	*S*. Typhimurium	(106)
*Helicteres isora*	Fruit	Aqueous	Antimutagenicity	22.77 ± 0.03 mg/ml	*S*. Typhimurium YG1024	(107)
*Aloysia triphylla*	Leaves	Chloramphenicol	Antibacterial	17.1 mg/ml	*S*. Typhimurium 245	(108)
*Cinnamomum zeylanicum*	Leaves, bark	Chloramphenicol	Antibacterial	0.63 mg/ml	*S*. Typhimurium 250	(108)
*Cymbopogon citratus*	Roots	Chloramphenicol	Antibacterial	17.9 mg/ml	*S*. Typhimurium 251	(108)
*Litsea cubeba*	Fruit	Chloramphenicol	Antibacterial	17.7 mg/ml	*S*. Typhimurium 252	(108)
*Mentha piperita*	Leaves, flower, stem, bark, and seeds	Chloramphenicol	Antibacterial	18.24 mg/ml	*S*. Typhimurium 258	(108)
*Syzygium aromaticum*	Dried flower buds, leaves, and stems	Chloramphenicol	Antibacterial	0.329 mg/ml	*S*. Typhimurium 261	(108)
*Curcuma* longa	Rhizomes	Chloroform	Antibacterial	10.7 ± 0.49 mg/ml	*S*. Typhimurim	(109)
*Morus alba*	Leaves	Aqueous	Antibacterial and antioxidant	10.51 ± 1.17 μg/ml	*S*. Typhimurium	(101)
*Salvia officinalis*	Leaves	Aqueous	Antibacterial	0.045 mg/ml	*S*. Typhimurium	(110)
*Flacourtia indica*	Bark	Aqueous	Anti-salmonella	12 mg/ml	*S*. Typhimurium	(111)
*Swartzia madagascariensis*	Leaves	Aqueous	Antibacterial	23 mg/ml	*S*. Typhimurium	(111)
*Ximenia caffra*	Leaves	Aqueous	Antibacterial	11 mg/ml	*S*. Typhimurium	(111)
*Diospyros mespiliformis*	Leaves	Aqueous	Inhibitory activity	25 mg/ml	*S*. Typhimurium ATCC 14028	(112)
*Brachychiton bidwillii*	Leaf	Aceton	Antibacterial	0.31 mg/ml	*S*. Typhimurium	(113)
*Loxostylis alata*	Leaf	Acetone	Antibacterial	0.08 ± 0.00 mg/ml	*S*. Typhimurium (ATCC 14028)	(114)

Plants	Plant part	Extract	Biological activity	MIC	Bacteria type	References
*Trema guineensis*	Leaves, stem-bark and roots	Ethanol	Anti-*Salmonella typhi*	24–33 mg/ml	MDR-*S*. Typhi strains	(115)
*Newbouldia laevis*	Leaf	Methanolic	Bactericidal activity	3.125 mg/ml	*S*. Typhimurium	(116)
*Cymbopogon flexuosus*	Herb grass	Ethanol	Antimicrobial	0.4/0.1 v/v	*S*. Typhimurium	(98)
*Lavandula hybrida reydova*	Flowering plant	Ethanol	Highest inhibitory effect	0.4/0.1 v/v	*S*. Typhimurium	(98)
*Eugenia caryophyllus*	Flower bud	Ethanolic	Antibacterial	0.1/0.025 v/v	*S*. Typhimurium	(98)
*Cinnamomum cassia*	Barks	Methanol	Inhibit the growth of *Salmonella*	0.025/0.013 v/v	*S*. Typhimurium SL 1344	(98)
*Satureja montana*	Flowering plant	Methanolic	Inactivate bacteria	0.05/0.013 v/v	*S*. Typhimurium	(98)
*Phyllanthus amarus*	Leaves	Ethanolic	Strong antibacterial activity	8.0 mm	*S*. Typhi	(117)
*Mimusops elengi*	Bark	Methanol	Anti-typhoid	4.6 ± 0.3 mg/ml	*S*. Typhimurium	(73)
*Acacia catechu*	Leaves	Methanol	Antibacterial	700 μg/ml	*S*. Typhi	(118)
*Aegle marmelos*	Fruits	Methanol	Strong inhibitory effect	1.25–10 mg/ml	*S*. Typhimurium	(119)
*Acalypha australis*	Leafs	Ethanol	Antidiarrheal	1 mg/ml	*S*. Typhi	(120)
*Fagraea fragrans*	Leaf, bark and twig	Methanolic	Antibacterial	500 μg/ml	*S*. Typhimurium	(121)
*Momordica balsamina*	Fruit	Ethanol	Bactericidal	600 μg/ml	MDR-*S*. Typhi strains	(122)
*Andrographis paniculata*	Leaf	Methanol	Antibacterial	500 μg/ml	*S*. Typhimurium	(103)
*Croton roxburghii*	Leaf	Methanol	Antibacterial	156 μg/ml	*S*. Typhimurium	(103)
*Vitex negundo*	Leaf	Methanol	Antibacterial	5,000 μg/ml	*S*. Typhimurium	(103)
*Combretum paniculatumand*	Leaves	Ethanolic	Anti-*Salmonella typhi*	5.3 *mg/ml*	MDR-*S*. Typhi strains	(123)
*Coriandrum sativum*	Roots	Ethanol	Antimicrobial	0.2/0.003 v/v	*S*. Typhi	(124)
*Acacia* nilotica	Bark	Phenol	Antibacterial	6.25 mg/ml	*S*. Typhimurium	(125)
*Elaeis guineensis*	Leaf	Methanol	Antibacterial	8.33 ± 0.33 mg/ml	*S*. Typhimurium	(126)
*Boehmeria platyphylla*	Root	Methanol	Antibacterial	7 ± 0.2 mm	*S*. Typhi	(127)
*Terminalia avicennioides*	Root	Ethanol	Bactericidal	12.5–25 mg/ml	MDR-*S*. Typhi strains	(128)
*S. aromaticum*	Flower buds	n-Hexane	Antibacterial	1.318 mg/ml	*S*. Typhimurium	(109)
*Picrorhiza kurroa*	Leaves	Hydro-alcoholic	Antibacterial	7.81 μg/ml	*S*. Typhimurium	(129)
*Syzygium cumini*	Pulp	Phenolic	Inhibitory activity	>0.78 mg/g	*S*. Typhimurium	(130)
*Petroselinum crispum*	Leaves	Ethanol	Antibacterial	47.62 μl/ml	*S*. Typhimurium	(110)
*Levisticum officinale*	Leaves	Ethanol	Antibacterial	47.62 μl/ml	*S*. Typhimurium	(110)
*Thymus vulgare*	Leaves	Hexanic	Antibacterial	0.56 μl/ml	*S*. Typhimurium	(110)
*Occimomum basilicum*	Leaf	Methanol	Antibacterial	22.68 μl/ml	*S*. Typhimurium	(110)
*Petroselinum crispum*	Leaves	Ethanol	Antibacterial	3.00 ± 2.65 mg/ml	*S*. Typhimurium TA98	(131)
*Petroselinum crispum*	Leaves	Ethanol	Antibacterial	2.00 ± 0.00 mg/ml	*S*. Typhimurium TA100	(131)
*Bauhinia holophylla*	Leaves	Hydro-alcoholic	Mutagenic	214 ± 24 mg/plate	*S*. Typhimurium TA 97a	(132)
*Kirkia wilmsii*	Leaf	Ethanol	Antibacterial	0.31 mg/ml	*S*. Typhimurium	(113)
*Noltea africana*	Leaf	Ethanol	Antibacterial	0.63 mg/ml	*S*. Typhimurium	(113)
*Protorhus longifolia*	Leaf	Methanol	Antibacterial	0.31 mg/ml	*S*. Typhimurium	(113)
*Carissa macrocarpa*	Leaf	Methanol	Antibacterial	0.31 mg/ml	*S*. Typhimurium	(113)
*Anacardium occidentale*	Leaf	Ethanolic	Inhibitory activity	12.5 mg/ml	*S*. Typhimurium ATCC 14028	(112)
*Daniellia oliveri*	Leaf	Hdrothanolic	Inhibitory activity	12.5 mg/ml	*S*. Typhimurium ATCC 14028	(112)
*Pterocarpus erinaceus*	Stem bark	Hydrothanolic	Inhibitory activity	25 mg/ml	*S*. Typhimurium ATCC 14028	(112)
*Ochrosia elliptica*	Leaves	Ethanolic	Antibacterial	3.9 μl/ml	*S*. Typhimurium	(133)
*Aloe barbadensis*	Leaf	Methanol	Antibacterial	4.5 μg/ml	*Salmonella enterica*	(134)
*Adhatoda vasica*	Leaf	Methanol	Antioxidant	9.5 μg/ml	*Salmonella enterica*	(134)
*Amaranthus hybridus*	Leaf	Methanol	Antibacterial	6 μg/ml	*Salmonella enterica*	(134)
*Loxostylis alata*	Leaf	Ethanol	Strong inhibition activity	0.31 ± 0.00 mg/ml	*Salmonella Enteritidis* (ATCC 13076)	(114)
*Loxostylis alata*	Leaf	Ethanol	Antibacterial	0.16 ± 0.00 mg/ml	*S*. Typhimurium	(114)
*Loxostylis alata*	Leaf	Methanol	Antimicrobial	0.12 ± 0.06 mg/ml	*S*. Typhimurium	(114)
*Cinnamomum zeylanicum*	Dried powder	Methanol	Antibacterial	24.57 ± 0.58 mm	*S*. Typhi	(135)

## Conclusion and future prospective

*Salmonella* species have been labeled environmental persisters, mostly because of their powerful biofilm forming capacity. Because of this, a long lasting and persistent colonization of people, animals and plants is typically occurring. It is essential to develop antibiotics alternatives as soon as possible due to growing concerns about the spread of superbugs and the slow development of new medications for both livestock and humans. However, it has been found that numerous plant extract and their isolated phytochemicals exhibit strong efficacy against organisms that cause foodborne diseases. Numerous phytochemicals have showed promise as bactericidal or antimicrobial agents that can enhance the effects of already available antibiotics. These phytochemicals have demonstrated the ability to block key mechanisms for the development of resistance, including cell permeability, replication machinery, efflux pumps, and other processes necessary for the pathogen’s survival and resistance. These phytochemicals have displayed great effectiveness against bacteria that are resistant to antibiotics when used in combination. The possibility of a synergistic interaction between phytochemicals and established or newly developed antimicrobial agents is an opportunity, while the development of novel plant based antibacterial products through combinatorial chemistry and computational design continues to be an exciting challenge. Future research should also concentrate on the toxicological and pharmacokinetic properties of plant extracts and phytochemicals.

## Author contributions

The author confirms being the sole contributor of this work and has approved it for publication.

## Conflict of interest

The author declares that the research was conducted in the absence of any commercial or financial relationships that could be construed as a potential conflict of interest.

## Publisher’s note

All claims expressed in this article are solely those of the authors and do not necessarily represent those of their affiliated organizations, or those of the publisher, the editors and the reviewers. Any product that may be evaluated in this article, or claim that may be made by its manufacturer, is not guaranteed or endorsed by the publisher.
